# Reference Genome Resource for the Citrus Pathogen *Phytophthora citrophthora*

**DOI:** 10.7150/jgen.89324

**Published:** 2024-01-01

**Authors:** Heike Möller, Beatrix Coetzee, Jan van Niekerk, Lindy Joy Rose

**Affiliations:** 1 Department of Plant Pathology, Stellenbosch University, Private Bag X1, Matieland 7602, South Africa.; 2 School for Data Sciences and Computational Thinking, Stellenbosch University, Private Bag X1, Matieland 7602, South Africa.; 3 Citrus Research International, P.O. Box 28, Nelspruit 1200, South Africa.

**Keywords:** *Phytophthora citrophthora*, oomycete, PacBio sequencing, *De novo* genome assembly, bioinformatics

## Abstract

*Phytophthora citrophthora* is an oomycete pathogen that infects citrus. Its occurrence in citrus-growing regions worldwide is considered a major contributor to crop losses. This study presents a high-quality genome resource for *P. citrophthora*, which was generated using PacBio HiFi long-read high-throughput sequencing technology. We successfully assembled a 48.5 Mb genome containing 16,409 protein-coding genes from high-quality reads. This marks the first complete genome assembly of *P. citrophthora*, providing a valuable resource to enhance the understanding of pathogenic behaviour and fungicide sensitivity of this destructive citrus pathogen.

## Introduction

*Phytophthora citrophthora* is the causal agent of root rot, gummosis, and branch canker in citrus trees, and brown rot in citrus fruit 1. This soil-borne pathogen was first described in 1906 by Smith and Smith. Along with *Phytophthora nicotianae*, it currently represents the most destructive *Phytophthora* species causing disease in citrus 2. This oomycete pathogen is widespread, causing significant tree and crop losses in all tropical and subtropical citrus regions worldwide 2. *P. citrophthora* was the first *Phytophthora* species reported in South African citrus 3 and has since then been reported in citrus orchards in various provinces of the country, including the Western Cape, Eastern Cape, Limpopo, and Mpumalanga 4. Despite *P. citrophthora* being classified as a threat, limited genetic information is available for this pathogen and no complete genome sequence has been published. A more comprehensive understanding of the molecular mechanisms of *P. citrophthora* will increase knowledge of its pathogenicity and aid in the improvement of current disease management practices. This communication presents a complete genome sequence to aid in this matter.

## Materials and Methods

The *P. citrophthora* isolate STE-U-9442 was isolated through soil baiting from a citrus nursery in the Eastern Cape Province of South Africa. It was grown in a 250 mL Erlenmeyer flask containing 100 mL potato dextrose broth (Difco^TM^). The culture was grown in a shaking incubator (± 120 rpm) at 27ºC for three to five days. After incubation, mycelia were harvested, washed with distilled water, and frozen at -80ºC. The frozen mycelia were ground to a fine powder in liquid nitrogen using a mortar and pestle. High-quality DNA (approximately 5,000 ng) was extracted from mycelia using a CTAB/PVP pre-extraction followed by the Qiagen DNeasy® Plant Mini Kit protocol (QIAGEN, Hilden, Germany). For the pre-extraction, 75 mg of ground tissue was added to a 2 mL Eppendorf tube containing sterilised glass beads. The samples were disrupted in a TissueLyser (QIAGEN, Hilden, Germany) twice for 30 sec at high speed, after which 1 mL of CTAB/PVP extraction buffer (prewarmed to 60ºC) was added to each sample (1.4 M NaCl, 2% CTAB (w/v), 0.1 M Tris (pH 8), 0.02 M EDTA (pH 8), 1% PVP (pH 8)). The PVP was added to the extraction buffer shortly before use. The samples were again disrupted twice for 30 sec at high speed. Then, 4 μL of proteinase K (10 mg/mL) was added to the solution and incubated at 60°C for 30 min, inverting tubes every 10 min. Thereafter, 3 μL of Rnase (100 mg/mL) was added to the solution and incubated at 60°C for 30 min, inverting tubes every 10 min. After centrifugation for 10 min at 13,000 rpm, the lysate was transferred to new 2 mL tubes and the samples were further treated according to the Qiagen DNeasy® Plant Mini Kit protocol from step 2 onwards. The quality of the DNA was determined using a NanoDrop spectrophotometer (ThermoFisher Scientific, Waltham, Massachusetts, USA), Qubit (ThermoFisher Scientific, Waltham, Massachusetts, USA), and BioAnalyzer (Agilent Technologies, Santa Clara, California, USA).

The genomic DNA library was constructed with a PacBio HiFi Library kit and was subjected to circular consensus sequencing on a PacBio Sequel II instrument by Macrogen (Seoul, South Korea) to generate HiFi reads. Preprocessing of reads was performed using SMRT Link software (Pacific Biosciences) whereby adapter sequences were removed and consensus sequences were generated through multiple passes around a circularised single DNA molecule (SMRTbell template). The Genome Assembly application, powered by the Improved Phase Assembler HiFi genome assembler (SMRT Link v11.0), was used to generate a *de novo* genome assembly using HiFi reads. Firstly, Pancake was used to overlap reads and the overlapped reads were phased using Nighthawk. Chimeras and duplicates were eliminated from the overlapped reads and a string graph was constructed, which resulted in the generation of primary contigs. Racon 5 was used to polish contigs with phased reads. Default parameters were used for all Genome Assembly application processes. Following assembly, the depth of coverage was determined by mapping the HiFi reads to the assembled contigs. During this step, contigs shorter than 1 kb were excluded. Genome completeness was evaluated with BUSCO (v5.3.0) 6 using lineage eukaryota_odb10.2019-11-20 (number of genomes: 70, number of BUSCOs: 255).

MAKER (v3.01.03) was used to predict gene location. Protein BLAST+ (v2.7.1+) was performed against UniProt Swiss-Prot (201806) to identify proteins using various databases, including GO 7, Interpro (v69.0) 8, Pfam (v31.0) 9, and EggNOG (v4.5.1) 10 to determine their function.

Using the HMMER and DIAMOND tools on the dbCAN server (https://bcb.unl.edu/dbCAN2/index.php) 11, 12, the predicted proteins of *P. citrophthora* were searched against the dbCAN, dbCAN-sub, and CAZy databases. Proteins selected by at least two of the searches were defined as carbohydrate-active enzymes (CAZymes).

Proteins with a signal peptide, predicted using signal version 6 13, but without transmembrane helices, predicted using TMHMM version 1.0.20 14, were defined as candidate effectors. These candidate effectors were subjected to screening with EffectorP version 3 15.

Protein sequences were subjected to a BLAST search (percent query coverage and identity cut-off of 35, E-value cut-off 1.0 x 10^-5^) against the Pathogen Host Interactions base 16, 17 to identify proteins associated with pathogenicity.

The contig corresponding to the mitochondrial (mtDNA) genome was identified based on similarity to the mitochondrial genome previously sequenced (Genbank accession number NC_067066.1). The genome was assembled and protein-coding genes were predicted with MFannot (https://megasun.bch.umontreal.ca/apps/mfannot/).

## Results and Discussion

Library sequencing resulted in 2,432,934 HiFi reads with an average read length of 10,393 bp. The final assembly product was a ~48.5 Mb genome, with coverage of 521 x. The genome consisted of 155 contigs with an N_50_ length of ~908.6 Kb (Table 1). Assessment of completeness showed that out of 255 BUSCO groups searched, the assembly of STE-U-9442 contained 233 complete and single-copy BUSCOs (91.37%), 6 complete and duplicated BUSCOs (2.35%), 7 fragmented BUSCOs (2.75%), and 9 missing BUSCOs (3.53%).

**Figure 1:**
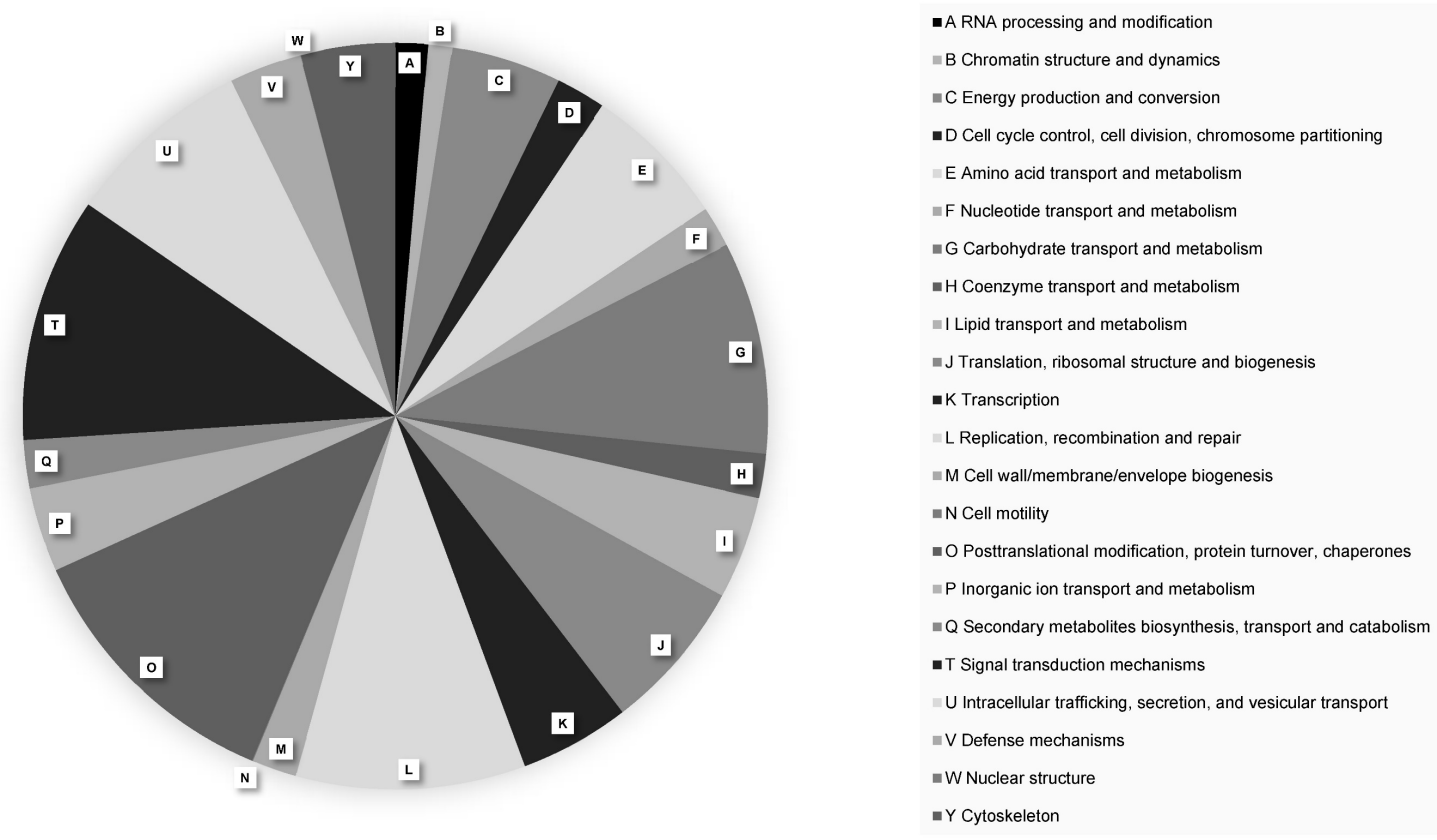
EggNOG functional protein classifications of Phytophthora citrophthora culture STE-U-9442. Different protein classes are indicated with A - Y.

A total of 16,409 protein-coding genes were predicted in the *P. citrophthora* STE-U-9442 genome (Table 2). The largest number of genes (630 genes) were annotated to have a function relating to the post-translational modification of proteins (Figure 1). Pathogens rely on protein changes to manipulate the plant host response, increase their activity during infection, and ultimately promote their survival 18. The high number of genes involved in post-translational modifications alludes to the complex interaction between *Phytophthora* and the citrus plant host and why this species is difficult to manage when infection is already established.

A total of 423 CAZymes were predicted for *P. citrophthora* STE-U-9442 (Table S1; File S1). As osmotrophs, *Phytophthora* species secrete hydrolytic enzymes that include CAZymes 19 and proteases that digest complex extracellular substrates, breaking down host cell wall components to establish infection and release nutrients. CAZymes have been predicted to play an important role in the disease cycle of many *Phytophthora* species 20-28.

In total, 713 effectors were predicted for *P. citrophthora* STE-U-9442, of which 420 is cytoplasmic and 293 is apoplastic (File S2). Oomycete pathogens, such as *Phytophthora*, secrete a large array of effector proteins to manipulate host immunity and facilitate infection 29, 30*.* Of the total 16,409 protein-coding genes in the genome, 1,157 were predicted to be pathogenicity related (Table S2).

The mitochondrial genome of *P*. *citrophthora* was assembled into a circular molecule of 37,510 bp with a 21.94% G+C content. It was predicted to encode 39 protein-coding genes, two ribosomal RNA genes, and 25 tRNA genes.

The full genome sequence of *P. citrophthora* will be essential for understanding the biology of this citrus pathogen, developing diagnostic tools for pathogen detection, identifying potential targets of disease control, and understanding the genetic evolution of this pathogen.

## Supplementary Material

Supplementary file 1.

Supplementary file 2.

Supplementary table 1.

Supplementary table 2.

## Figures and Tables

**Table 1:** Genome assembly statistics of Phytophthora species with available whole genome sequences on FungiDB, including Phytophthora citrophthora culture STE-U-9442 from this study.

Phytophthora species	Total length (basepairs)	N_50_ (basepairs)	Number of contigs	Number of BUSCOs	Number of protein coding genes
P. capsici strain LT1534	94 176 027	485 876	782	246	23 373
P. cinnamomi GKB4	109 702 272	1 187 988	133	252	19 981
P. cinnamomi var. cinnamomi CBS 144.22	77 967 402	264 472	1 314	240	26 131
P. infestans T30-4	228 543 505	1 588 622	4 921	249	17 797
P. palmivora var. palmivora strain sbr112.9	107 772 931	6 694	24 809	206	24 674
P. parasitica INRA-310	82 389 172	888 348	708	245	23 121
P. plurivora AV1007	40 441 201	48 620	1 897	255	-
P. ramorum strain Pr102	66 652 401	308 042	2 576	249	15 492
P. sojae strain P6497	82 597 641	7 609 242	82	256	26 489
P. citrophthora STE-U-9442 (this study)	48 478 215	908 581	155	233	16 409

**Table 2:** Genome characteristics of Phytophthora citrophthora culture STE-U-9442

Number of genes	20170
Number of CDS	16409
Average CDS (aa)	498
Average mRNA (bp)	1503
Average exons per gene	2
Number of exons	39348
Average exon (bp)	627
Number of introns	22939
Average intron (bp)	180
Number of tRNA	3703
Number of rRNA	64
Number of coding genes annotated with	
GO	6838
InterPro	9106
Pfam	8901
EggNOG	14949

## References

[1] Aglave B (2018). Citrus. In: Handbook of Plant Disease Identification and Management. Boca Raton: CRC Press.

[2] Erwin DC, Ribeiro OK (1996). Phytophthora diseases worldwide. St. Paul, USA: APS Press.

[3] Doidge EM (1920). Brown rot in citrus fruits (Phythiacystis citrophthora (R. and E. Sm.). Journal of the Department of Agriculture, Union of South Africa.

[4] Meitz-Hopkins JC, Pretorius MC, Spies CFJ (2013). Phytophthora species distribution in South African citrus production regions. Journal of Plant Pathology.

[5] Vaser R, Sović I, Nagarajan N (2017). Fast and accurate de novo genome assembly from long uncorrected reads. Genome Res.

[6] Manni M, Berkeley MR, Seppey M (2021). BUSCO Update: Novel and Streamlined Workflows along with Broader and Deeper Phylogenetic Coverage for Scoring of Eukaryotic, Prokaryotic, and Viral Genomes. Molecular Biology and Evolution.

[7] Harris MA, Clark J, Ireland A (2004). The Gene Ontology (GO) database and informatics resource. Nucleic Acids Research.

[8] Paysan-Lafosse T, Blum M, Chuguransky S (2023). InterPro in 2022. Nucleic Acids Research.

[9] Mistry J, Chuguransky S, Williams L (2021). Pfam: The protein families database in 2021. Nucleic Acids Research.

[10] Huerta-Cepas J, Szklarczyk, D, Heller D (2019). eggNOG 5.0: a hierarchical, functionally and phylogenetically annotated orthology resource based on 5090 organisms and 2502 viruses. Nucleic Acids Research.

[11] Yin Y, Mao X, Yang J (2012). dbCAN: a web resource for automated carbohydrate-active enzyme annotation. Nucleic Acids Research.

[12] Zhang H, Yohe T, Huang L (2018). dbCAN2: a meta server for automated carbohydrate-active enzyme annotation. Nucleic Acids Research.

[13] Teufel F, Almagro Armenteros JJ, Johansen AR (2022). SignalP 6.0 predicts all five types of signal peptides using protein language models. Nat Biotechnol.

[14] Hallgren J, Tsirigos KD, Pedersen MD (2022). DeepTMHMM predicts alpha and beta transmembrane proteins using deep neural networks. bioRxiv.

[15] Sperschneider J, Dodds PN (2022). EffectorP 3.0: Prediction of Apoplastic and Cytoplasmic Effectors in Fungi and Oomycetes. MPMI.

[16] Urban M, Cuzick A, Seager J (2020). PHI-base: the pathogen-host interactions database. Nucleic Acids Research.

[17] Urban M, Cuzick A, Seager J (2022). PHI-base in 2022: a multi-species phenotype database for pathogen-host interactions. Nucleic Acids Research.

[18] Retanal C, Ball B, Geddes-McAlister J (2021). Post-translational modifications drive success and failure of fungal-host interactions. JoF.

[19] Lombard V, Golaconda Ramulu H, Drula E (2014). The carbohydrate-active enzymes database (CAZy) in 2013. Nucleic Acids Research.

[20] Blackman LM, Cullerne DP, Hardham AR (2014). Bioinformatic characterisation of genes encoding cell wall degrading enzymes in the Phytophthora parasitica genome. BMC Genomics.

[21] Blackman LM, Cullerne DP, Torreña P (2015). RNA-seq analysis of the expression of genes encoding cell wall degrading enzymes during infection of lupin (Lupinus angustifolius) by Phytophthora parasitica. PLOS ONE.

[22] Brouwer H, Coutinho PM, Henrissat B (2014). Carbohydrate-related enzymes of important Phytophthora plant pathogens. Fungal Genetics and Biology.

[23] Grams N, Ospina-Giraldo M (2019). Increased expression of Phytophthora sojae genes encoding membrane-degrading enzymes appears to suggest an early onset of necrotrophy during Glycine max infection. Fungal Genetics and Biology.

[24] Hu Y, He Z, Kang Y (2022). Identification of a C2H2 transcription factor (PsCZF3) associated with RxLR effectors and carbohydrate-active enzymes in Phytophthora sojae based on WGCNA. Journal of Fungi.

[25] McGowan J, O'Hanlon R, Owens RA (2020). Comparative genomic and proteomic analyses of three widespread Phytophthora species: Phytophthora chlamydospora, Phytophthora gonapodyides and Phytophthora pseudosyringae. Microorganisms.

[26] Thorpe P, Vetukuri RR, Hedley PE (2021). Draft genome assemblies for tree pathogens Phytophthora pseudosyringae and Phytophthora boehmeriae. G3 Genes|Genomes|Genetics.

[27] Toljamo A, Blande D, Munawar M (2019). Expression of the GAF sensor, carbohydrate-active enzymes, elicitins, and RXLRs differs markedly between two Phytophthora cactorum isolates. Phytopathology.

[28] Yang M, Duan S, Mei X (2018). The Phytophthora cactorum genome provides insights into the adaptation to host defense compounds and fungicides. Sci Rep.

[29] Andronis CE, Jacques S, Lipscombe R (2022). Comparative sub-cellular proteome analyses reveals metabolic differentiation and production of effector-like molecules in the dieback phytopathogen Phytophthora cinnamomi. Journal of Proteomics.

[30] Gao RF, Wang JY, Liu KW (2021). Comparative analysis of Phytophthora genomes reveals oomycete pathogenesis in crops. Heliyon.

